# Class I HDACs specifically regulate E‐cadherin expression in human renal epithelial cells

**DOI:** 10.1111/jcmm.12919

**Published:** 2016-07-15

**Authors:** Sin Y. Choi, Hae J. Kee, Thomas Kurz, Finn K. Hansen, Yuhee Ryu, Gwi R. Kim, Ming Q. Lin, Li Jin, Zhe H. Piao, Myung H. Jeong

**Affiliations:** ^1^ Heart Research Center of Chonnam National University HospitalGwangjuKorea; ^2^ Institute of Pharmaceutical and Medicinal ChemistryHeinrich Heine University DüsseldorfDüsseldorfGermany; ^3^ Jilin Hospital Affiliated with Jilin UniversityJilinChina; ^4^ The Second Hospital of Jilin UniversityChangchunChina

**Keywords:** class I HDAC, epithelial‐mesenchymal transition (EMT), renal fibrosis, HK‐2 cells, E‐cadherin

## Abstract

Epithelial‐mesenchymal transition (EMT) and renal fibrosis are closely involved in chronic kidney disease. Inhibition of histone deacetylase (HDAC) has an anti‐fibrotic effect in various diseases. However, the pathophysiological role of isoform‐specific HDACs or class‐selective HDACs in renal fibrosis remains unknown. Here, we investigated EMT markers and extracellular matrix (ECM) proteins in a human proximal tubular cell line (HK‐2) by using HDAC inhibitors or by knockdown of class I HDACs (HDAC1, 2, 3 and 8). Trichostatin A (TSA), MS275, PCI34051 and LMK235 inhibited ECM proteins such as collagen type I or fibronectin in transforming growth factor β1 (TGF‐β1)‐induced HK2 cells. However, restoration of TGF‐β1‐induced E‐cadherin down‐regulation was only seen in HK‐2 cells treated with TSA or MS275, but not with PCI34051, whereas TGF‐β1‐induced N‐cadherin expression was not affected by the inhibitors. ECM protein and EMT marker levels were prevented or restored by small interfering RNA transfection against HDAC8, but not against other class I HDACs (HDAC1, 2 and 3). E‐cadherin regulation is mediated by HDAC8 expression, but not by HDAC8 enzyme activity. Thus, class I HDACs (HDAC1, 2, 3 and 8) play a major role in regulating ECM and EMT, whereas class IIa HDACs (HDAC4 and 5) are less effective.

## Introduction

Fibrosis is characterized by excessive accumulation of extracellular matrix (ECM) proteins and is associated with various pathological diseases including chronic kidney disease (CKD) 1. A recent systematic review showed that CKD is associated with high risk for all‐cause and cardiovascular mortality 2. Fibrosis is stimulated by many factors including inflammation, hypertension and oxidative stress 3. Transforming growth factor β1 (TGF‐β1) is a well‐known pro‐fibrotic cytokine that plays an important role in renal fibrosis 4.

Epithelial‐mesenchymal transition (EMT) contributes to embryonic development, tissue fibrosis, cancer progression and metastasis 5. EMT of proximal tubular cells plays an important role in renal fibrosis and production of ECM components 6, 7. EMT is defined as the loss of epithelial cell markers and gain of mesenchymal markers. The expression of cadherin is switched from E‐cadherin to N‐cadherin during EMT with spindle‐shaped morphological changes.

Histone deacetylase (HDAC) inhibitors have beneficial effects on the modulation of proliferation, differentiation, survival, inflammation, apoptosis and cell migration in various diseases 8, 9. HDAC inhibitors have been reported to block renal fibrosis in a murine model of unilateral ureteral obstruction (UUO) 10. On the contrary, increased EMT was observed in colon carcinoma cells on using the HDAC inhibitors TSA and valproic acid 11. HDACs are classified into four subfamilies. Each of the HDAC isoforms plays a different role in various tissues. Recent studies show that HDAC1, HDAC2 and HDAC6 are required for TGF‐β1‐induced EMT 12, 13, 14. However, the specific HDAC that plays a critical role in controlling EMT and fibrosis *in vivo* and *in vitro* remains unclear.

In this study, we investigated the effect of various HDAC inhibitors (TSA, MS275, PCI34051 and LMK235) on TGF‐β1‐induced EMT and its regulatory mechanism in human renal proximal epithelial cells (HK‐2). Our findings indicate that class I HDACs (HDAC1, 2 and 3) and class II HDACs (HDAC4 and HDAC5) are implicated in the EMT process in HK‐2 cells. Small interfering RNA (siRNA) experiments demonstrate that HDAC8 expression, but not HDAC8 enzyme activity, plays an important role in mediating EMT‐related fibrosis.

## Materials and methods

### Materials

Recombinant human TGF‐β1 (PHG9204) was purchased from Invitrogen (Waltham, MA, USA). Trichostatin A (TSA) was purchased from Sigma (T8552, St. Louis, MO, USA). MS275 and PCI34051 were purchased from Selleckchem (S1053 and S2012, respectively, Burlington, NC, USA). LMK235 was obtained from Prof. Thomas Kurz (Heinrich‐Heine Universität Düsseldorf, Germany) and synthesized according to a previously published protocol 15. To rule out the siRNA off‐target effect, we used siRNAs from two companies (Bioneer and Santa Cruz). siRNAs against HDAC1, HDAC2, and HDAC3 were purchased from Bioneer (Gyeonggi‐do, South Korea) and siRNAs against HDAC2, HDAC3, and HDAC8 were purchased from Santa Cruz Biotechnology (Dallas, TX, USA); HDAC2 siRNA (sc‐29345), HDAC3 siRNA (sc‐35538), and HDAC8 siRNA (sc‐35548).

### Antibodies

Antibodies against E‐cadherin (sc‐7870), HDAC1 (sc‐7872), HDAC2 (sc‐7899), HDAC3 (sc‐11417), HDAC8 (sc‐11405), alpha smooth muscle actin (α‐SMA, sc‐130617) and β‐actin (sc‐4778) were purchased from Santa Cruz Biotechnology. Anti‐Collagen type I was obtained from Abcam (Cambridge, MA, USA). Anti‐N‐cadherin (#14215) was purchased from Cell Signaling Technology (Danvers, MA, USA) and anti‐Fibronectin (MA5‐11981) was purchased from Thermo Fisher Scientific (Waltham, MA, USA).

### Cell culture

Human kidney proximal tubule epithelial cell line HK2 was obtained from the Korean Cell Line Bank (Seoul, South Korea). HK2 cells were maintained in RPMI medium supplemented with 10% fetal bovine serum (FBS). The cells used in the experiments were grown to approximately 80% confluence. To test the effect of HDAC inhibitors on ECM proteins and EMT markers, the cells were serum starved overnight and co‐treated with TGF‐β1 and HDAC inhibitors for the indicated time points.

### Cell viability assay

Cell viability was determined by the MTT [3‐(4,5‐dimethylthiazol‐2‐yl)‐2,5‐diphenyl tetrazolium bromide] assay. HK2 cells were seeded onto 24‐well culture dishes at a density of 40,000 cells/ml and were maintained with RPMI containing 10% FBS. Cells were treated with HDAC inhibitors (TSA, MS275, PCI34051 and LMK235) or vehicle for 24 hrs at the indicated concentrations. After 24 hrs, MTT was added to the medium and the absorbance was measured at 570 nm.

### siRNA transfection

HK2 cells were seeded into 12‐well culture dishes at a density of 60,000 cells/ml. The cells were then transfected with control siRNA, or siRNA against HDAC1, HDAC2, HDAC3 or HDAC8 performed with RNAiMax reagents (Thermo Fisher Scientific). All siRNAs were used at a concentration of 100 nM. To verify HDAC knockdown, we performed real‐time polymerase chain reaction (RT‐PCR) 1 day after transfection. To investigate the effect of HDAC siRNA on ECM or EMT, the transfected cells were serum starved overnight and then incubated with TGF‐β1 for 24 hrs.

### Western blot analysis

Western blots were performed as described previously 16. Cells lysates were prepared in RIPA buffer (150 mM NaCl, 1% Triton X‐100, 1% sodium deoxycholate, 50 mM Tris‐HCl, pH 7.5, 2 mM EDTA, 1 mM PMSF, 1 mM DTT, 1 mM Na_3_VO_4_, 5 mM NaF) containing protease inhibitors. Proteins were separated by 8% SDS‐PAGE and were then transferred to polyvinylidene difluoride membranes. The membranes were probed with the indicated antibodies and developed using Immobilon Western Detection Reagents (Millipore, Billerica, MA, USA). Protein expression was quantified using Bio‐ID software (Vilber Lourmat, Germany).

### Immunocytochemistry

HK2 cells were seeded on coverslips in 12‐well culture dishes at a density of 60,000 cells/ml. The cells were serum starved overnight and then treated with HDAC inhibitors or vehicle in the presence of TGF‐β1 for 24 hrs. Cells were then fixed with 70% methanol for 30 min, permeabilized and blocked with 4% normal goat serum for 1 hr. These cells were incubated with the E‐cadherin antibody (1:100) overnight at 4°C and were then incubated with Alexa Fluor 488‐conjugated rabbit anti‐mouse IgG antibody (1:400) for 1 hr. The stained cells were visualized and imaged by fluorescence microscopy (Nikon, Tokyo, Japan).

### Quantitative real time polymerase chain reaction

Total RNA was extracted using TRIzol reagent (Invitrogen Life Technologies, Waltham, MA, USA), and 1 μg of RNA was used for the reverse transcription reaction with TOPscript RT DryMIX (Enzynomics, Daejeon, South Korea). Quantification of mRNA was determined using a SYBR Green PCR kit (Enzynomics). Relative expression levels of the indicated genes were compared with GAPDH expression using the 2−^ΔΔct^ method 17. PCR was performed using the following primers: for HDAC1, sense, 5′‐ACGAGTCCTATGAGGCCATTT‐3′, and antisense, 5′‐CACTTGGCGTGTCCTTTGATA‐3′; for HDAC2, sense, 5′‐CATAAAGCCACTGCCGAAGAA‐3′, and antisense, 5′‐TCCATCAAACGCTGGACAATC‐3′; for HDAC3, sense, 5′‐GGTGTCCTTCCACAAATACGG‐3′, and antisense, 5′‐GGCTGGAAAAGGTGCTTGTAA‐3′; for HDAC8, sense, 5′‐AGTGGGAATTGGCAAGTGTC‐3′, and antisense, 5′‐CCAGCACATAATCAGGACCA‐3′; for GAPDH, sense, 5′‐GAAGGTGAAGGTCGGAGTCA‐3′, and antisense, 5′‐GACAAGCTTCCCGTTCTCAG‐3′.

### Statistical analysis

Statistical analysis was performed either by the Student's *t*‐test or by one‐way anova followed by the Bonferroni *post hoc* test using GraphPad Prism version 5.0 (GraphPad Software Inc., La Jolla, CA, USA). Data are presented as means ± SD. *P* < 0.05 was considered as statistically significant.

## Results

### TGF‐β1 induces ECM protein expression and EMT in HK2 cells

**Figure 1:**
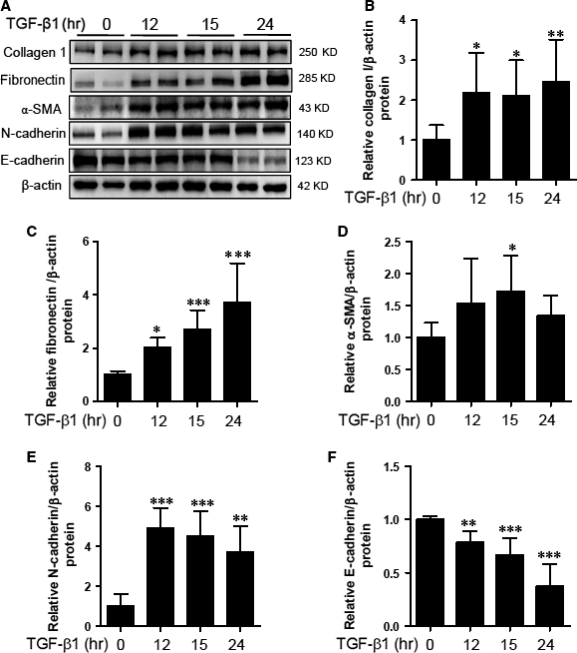
Extracellular matrix (ECM) proteins and epithelial‐mesenchymal transition (EMT) markers are induced by TGF‐β1. HK‐2 cells were incubated with TGF‐β1 (5 ng/ml) for the indicated time points. (**A**) Representative immunoblots. (**B–F**) Fibronectin, collagen type I, α‐SMA, N‐cadherin and E‐cadherin protein expression was quantified by densitometry. Data are expressed as mean ± SD of six independent experiments. *P < 0.05, **P < 0.01, and ***P < 0.001 compared with untreated cells.

TGF‐β1 is a well‐known key mediator of fibrosis including EMT 18. To identify whether TGF‐β1 could induce the expression of ECM proteins and EMT markers, we performed Western blot analysis. Human epithelial HK2 cells were treated with TGF‐β1 at the indicated time points. As shown in Figure 1A–C, ECM proteins such as collagen type I and fibronectin were induced by TGF‐β1 stimulus. Fibrosis is associated with the activation of myofibroblasts showing expression of α‐SMA 19. TGF‐β1 increased α‐SMA protein levels (Fig. 1D). Fibrosis is characterized by the switch in cadherin expression from E‐cadherin to N‐cadherin 20, 21. TGF‐β1 increased N‐cadherin and decreased E‐cadherin protein expression in HK2 cells (Fig. 1E and F).

### Non‐specific pharmacological inhibition of class I and class II HDACs restores TGF‐β1‐mediated E‐cadherin expression in HK2 cells

**Figure 2:**
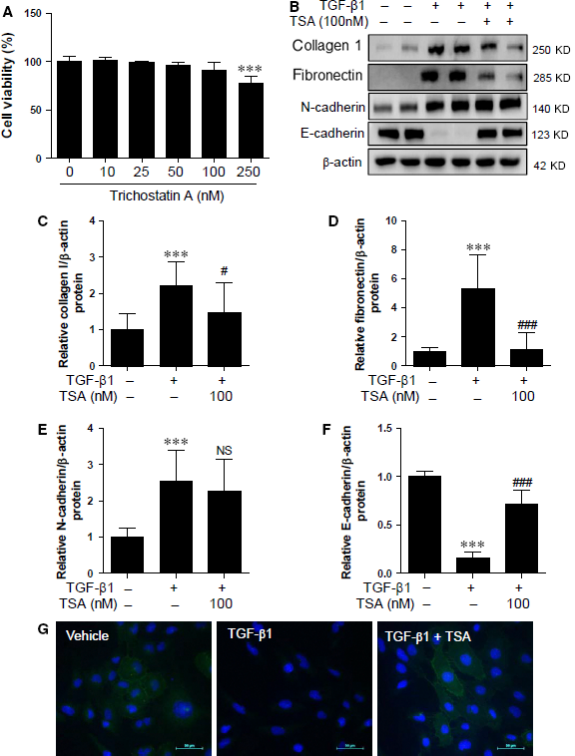
TSA regulates TGF‐β1‐mediated ECM proteins and E‐cadherin in HK2 cells. (**A**) HK2 cells were treated with increasing concentrations of TSA (0, 10, 25, 50 100 and 250 nM) for 24 hrs and cell viability was measured by the MTT assay. (**B**) HK2 cells were co‐treated with TGF‐β1 (5 ng/ml) and TSA (100 nM). ECM proteins and EMT markers were determined using Western blot analysis. Representative immunoblots are shown. β‐Actin was used as the loading control. (**C–F**) Quantification was performed by densitometry for at least four independent experiments. (**G**) Representative immunofluorescence images for E‐cadherin in HK2 cells. Immunofluorescence staining was performed using anti‐E‐cadherin antibody and DAPI was used for staining cell nuclei. Merged images are shown. Scale bar represents 50 μm. ***P < 0.001 compared with untreated cells. ^#^ *P* < 0.05 and ^###^ *P* < 0.001 compared with TGF‐β1‐treated cells. NS, not significant.

We tested the cytotoxicity of TSA, a non‐specific HDAC inhibitor, in HK2 cells by using the MTT assay (Fig. 2A). We used the optimal concentration of TSA (100 nM) that did not cause cytotoxicity. TSA decreased the protein expression of collagen type I induced by TGF‐β1 (Fig. 2B and C). Western blot analysis clearly demonstrated that TSA completely inhibited TGF‐β1‐induced fibronectin protein expression (Fig. 2B and D).

We next determined whether TSA could regulate EMT in HK2 cells. N‐cadherin and E‐cadherin are markers of mesenchymal and epithelial features respectively. TSA did not affect TGF‐β1‐induced N‐cadherin protein levels (Fig. 2B and E). Treatment with TGF‐β1 significantly decreased E‐cadherin protein expression, which was considerably restored by TSA (Fig. 2B and F). This result was further confirmed by fluorescence immunocytochemistry (ICC) for E‐cadherin. ICC showed that E‐cadherin expression was localized to the cell membrane in the basal state and that its expression was decreased in response to TGF‐β1. This reduced E‐cadherin expression could be recovered by TSA (Fig. 2G).

### Pharmacological inhibition of class I HDAC (1, 2, 3) restores TGF‐β1‐mediated E‐cadherin down‐regulation in HK2 cells

**Figure 3:**
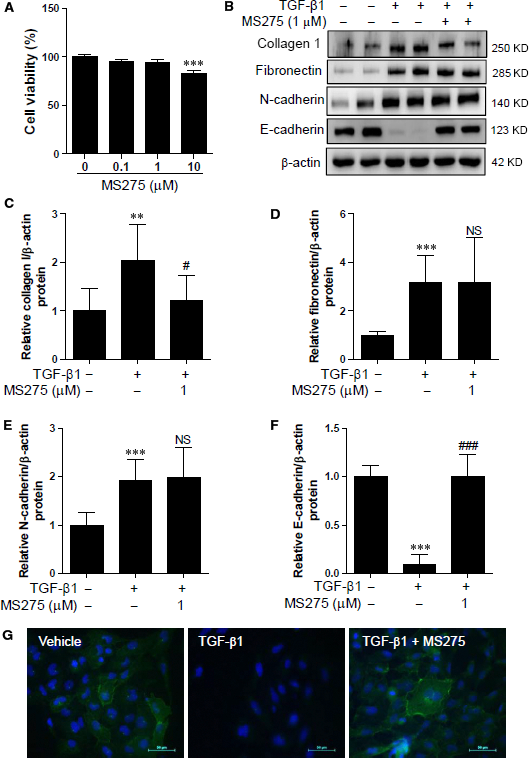
MS275 regulates TGF‐β1‐mediated ECM proteins and E‐cadherin in HK2 cells. (**A**) HK2 cells were incubated with increasing concentrations of MS275 (0, 0.1, 1 and 10 μM) for 24 hrs and cell viability was determined. (**B**) HK2 cells were co‐treated with TGF‐β1 (5 ng/ml) and MS275 (1 μM). Proteins from cell lysates were subjected to SDS‐PAGE. Representative Western blot images are shown. β‐Actin was used as the loading control. (**C–F**) Quantification was performed by densitometry for at least four independent experiments. (**G**) Representative immunofluorescence images for E‐cadherin in HK2 cells. Immunofluorescence staining was performed using anti‐E‐cadherin antibody and cell nuclei were stained with DAPI. Merged images are shown. Scale bar represents 50 μm. **P < 0.01 and ***P < 0.001 compared with untreated cells. ^#^ *P* < 0.05 and ^###^ *P* < 0.001 compared with TGF‐β1 treated cells. NS, not significant.

Based on the MTT assay results, we used MS275 at 1 μM without causing cytotoxicity (Fig. 3A). MS275 is a strong inhibitor of HDAC1, HDAC2 and HDAC3, but not of HDAC8. Treatment of HK2 cells with MS275 effectively ameliorated TGF‐β1‐induced expression of collagen type I protein, but not of fibronectin (Fig. 3B–D). Similar to TSA, MS275 did not affect TGF‐β1‐induced N‐cadherin expression (Fig. 3B and E). However, MS275 completely restored the E‐cadherin reduction induced by TGF‐β1 (Fig. 3B and F). ICC demonstrated that E‐cadherin was localized to the cell membranes under normal conditions. Decreased expression of E‐cadherin mediated by TGF‐β1 was recovered by MS275 (Fig. 3G).

### HDAC8‐selective inhibition does not affect TGF‐β1‐mediated EMT in HK2 cells

**Figure 4:**
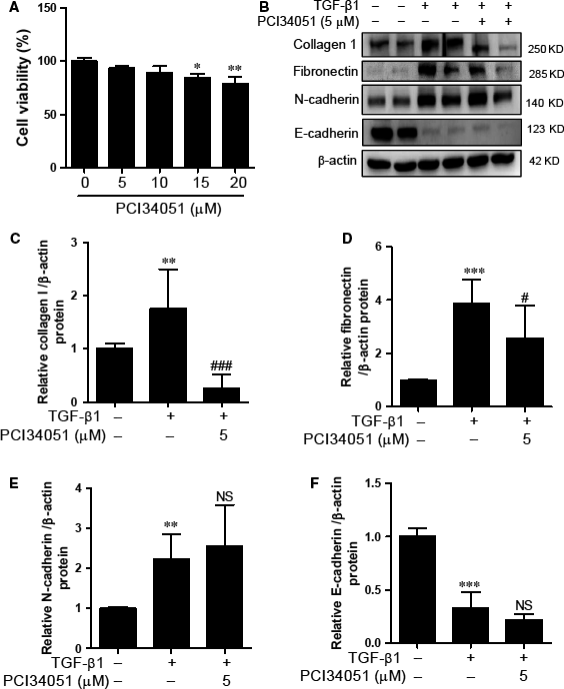
PCI34051 did not affect TGF‐β1‐mediated EMT markers in HK2 cells. (**A**) Cytotoxicity of PCI34051 in HK2 cells. (**B**) HK2 cells were co‐treated with TGF‐β1 (5 ng/ml) and PCI34051 (5 μM) for 24 hrs. Western blot analysis was performed using collagen type I (collagen 1), fibronectin, N‐cadherin, and E‐cadherin antibodies. β‐Actin was used as the loading control. (**C–F**) Quantification was performed by densitometry for at least four independent experiments. **P < 0.01 and ***P < 0.001 compared with untreated cells. ^#^ *P* < 0.05 and ^###^ *P* < 0.001 compared with TGF‐β1 treated cells. NS, not significant.

HDAC8 belongs to class I HDACs. We investigated whether HDAC8 could be involved in EMT‐related fibrosis. We used PCI34051, a selective HDAC8 inhibitor, which effectively inhibits HDAC8, but not HDAC1, HDAC2, HDAC3 or class II HDACs (HDAC6 and HDAC10). PCI34051 did not show any cytotoxicity at a concentration of 5 μM (Fig. 4A). PCI34051 inhibited ECM proteins including collagen type I and fibronectin (Fig. 4B–D), but did not affect the expression of N‐cadherin and E‐cadherin (Fig. 4B, E and F).

### HDAC4/5‐selective inhibition shows partial reversal of E‐cadherin expression in HK2 cells

**Figure 5:**
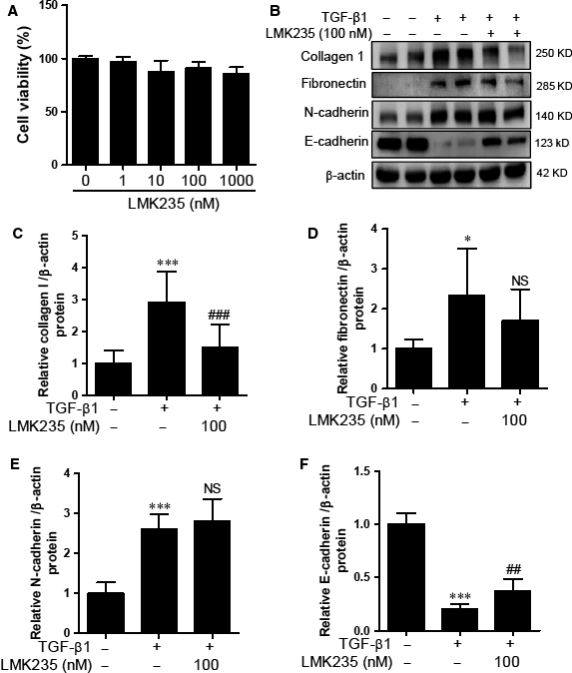
LMK235 did not affect TGF‐β1‐mediated ECM proteins and EMT markers in HK2 cells. (**A**) Cytotoxicity of LMK235 in HK2 cells. (**B**) HK2 cells were co‐treated with TGF‐β1 and LMK235 for 24 hrs. Western blot analysis was performed using collagen I, fibronectin, N‐cadherin, and E‐cadherin antibodies. β‐Actin was used as the loading control. (**C–F**) Quantification was performed by densitometry for at least four independent experiments. *P < 0.05 and ***P < 0.001 compared with untreated cells. ^##^ *P* < 0.01 and ^###^ *P* < 0.001 compared with TGF‐b1 treated cells. NS, not significant.

We evaluated the anti‐fibrotic effect of LMK235, which is a class IIa‐selective HDAC inhibitor with high potency against HDAC4 and HDAC5. Based on the results from the MTT assay and the isoform profile of LMK235 15, we decided to use a concentration of 100 nM of LMK235. LMK235 decreased TGF‐β1‐induced collagen type I expression in HK2 cells (Fig. 5B–D). However, LMK235 did not reduce TGF‐β1‐induced N‐cadherin protein levels in HK2 cells (Fig. 5B and E). Western blot analysis showed that LMK235 treatment partially recovered the E‐cadherin expression down‐regulated by TGF‐β1 in HK2 cells (Fig. 5B and F).

### Knockdown of HDAC8 attenuates ECM proteins and restores E‐cadherin expression in HK2 cells

**Figure 6:**
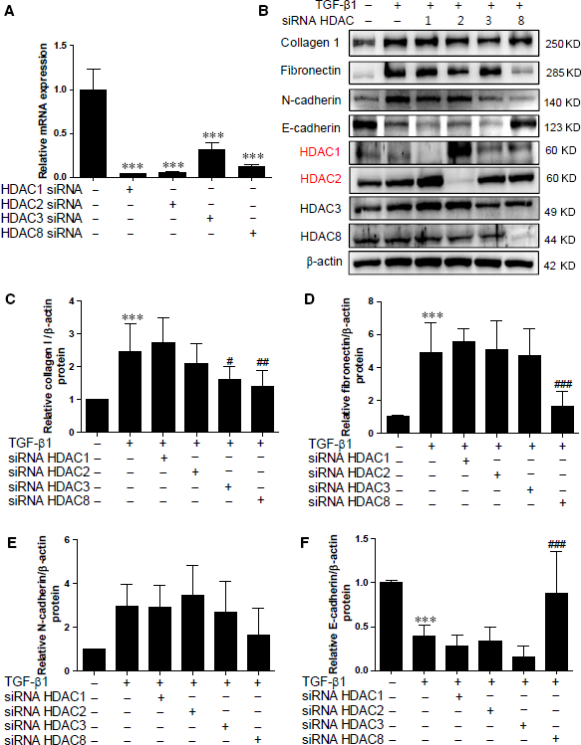
Knockdown of HDAC8 blocks ECM proteins and EMT markers induced by TGF‐β1. (**A**) HK2 cells were transfected with siRNA against class I HDACs (HDAC1, 2, 3 and 8) or siRNA control and then incubated with TGF‐β1 for 24 hrs. ***P < 0.001 compared with siRNA control. (**B**) Representative immunoblots for ECM proteins, EMT markers and class I HDACs. (**C–F**) Quantification was performed by densitometry for at least six independent experiments. ***P < 0.001 compared with siRNA control. ^#^ *P* < 0.05, ^##^ *P* < 0.01 and ^###^ *P* < 0.001 compared with TGF‐β1‐treated siRNA control cells.

To determine whether the knockdown of class I HDACs could affect ECM proteins and EMT markers, HK2 cells were transfected with siRNA against HDAC1, HDAC2, HDAC3, HDAC8 or control siRNA and were then incubated with TGF‐β1 for 24 hrs. We found that each of the siRNAs against class I HDACs effectively reduced the endogenous HDAC mRNA levels (Fig. 6A). As shown in Figure 6B, siRNA transfection significantly reduced the protein expression of class I HDACs in HK2 cells. In terms of HDAC levels, siRNA against HDAC1 increased HDAC2 protein levels whereas siRNA against HDAC2 enhanced HDAC1 protein levels (Fig. 6B). However, siRNA against HDAC8 did not induce the expression of other class I HDACs. Unlike the pharmacological HDAC inhibitors, HDAC1 and HDAC2 siRNA did not reduce ECM protein levels or restore the EMT markers (N‐cadherin and E‐cadherin). HDAC3 knockdown attenuated TGF‐β1‐induced collagen type I protein expression (Fig. 6B and C), but it did not affect the EMT markers (Fig. 6B, E and F). The most interesting finding was that siRNA against HDAC8 inhibited collagen type I and fibronectin expression induced by TGF‐β1 in HK2 cells. HDAC8 knockdown showed the tendency of reducing N‐cadherin expression in HK2 cells treated with TGF‐β1 (Fig. 6B and E). Moreover, HDAC8 knockdown considerably restored the E‐cadherin down‐regulated by TGF‐β1 (Fig. 6B and F). To rule out the siRNA off‐target effect, we performed siRNA transfection using other siRNAs including at least three target‐specific sites against HDAC2 or HDAC3. As addressed above, we observed similar results with both sets of siRNA (Figs S1 and S2).

## Discussion

In the present study, we demonstrated that chemical inhibitors of class I HDACs are more important for the regulation of the process of renal fibrosis than class II‐selective HDAC inhibitors. We first found that human kidney epithelial cells (HK2 cells) are a good cell line for the *in vitro* study of EMT. TGF‐β1 increased collagen type I, fibronectin and N‐cadherin expression but decreased E‐cadherin expression. These results indicate that TGF‐β1‐induced EMT and fibrosis were well induced in HK2 cells.

In this study, we used several class‐selective HDAC or isoform‐selective HDAC inhibitors in the process of fibrosis or EMT. We conclude that class I HDACs (HDAC1, 2, 3 and 8) and class IIa HDACs (HDAC4 and HDAC5) are involved in the accumulation of ECM proteins. These results are derived from the findings that TSA, MS275, PCI34051 and LMK235 significantly suppressed collagen type I or fibronectin expression.

LMK235 is a selective HDAC4/5 inhibitor. TSA has been reported to inhibit class I HDACs and class II HDACs 22. MS275, a synthetic benzamide, has a weaker HDAC inhibitory activity than TSA. Furthermore, MS275 did not affect the enzymatic activity of class II HDACs. Consistent with our findings, TSA and MS275 inhibited the expression of collagen I and fibronectin in a mouse model of UUO 10, 23. To our knowledge, this is the first evidence showing that PCI34051, an HDAC8‐selective inhibitor, cannot attenuate the expression of ECM proteins. This finding suggests that HDAC8 enzyme activity is not required for ECM production. Recently, we reported that tubastatin A, an HDAC6‐selective inhibitor, suppresses renal fibrosis 24. Therefore, we cannot rule out the possible relevance of HDAC6, 7 and 9 in the accumulation of ECM proteins in HK2 cells.

EMT is involved in the process of renal fibrosis 25. The four HDAC inhibitors used in this study did not show complete inhibition of N‐cadherin expression. At present, the reason owing to which HDAC inhibitors failed to inhibit N‐cadherin expression is not known. One possible explanation could be that HDAC inhibitors favourably act to block E‐cadherin loss when cells are subjected to TGF‐β1.

However, two HDAC inhibitors, TSA and MS275, almost completely restored TGF‐β1‐mediated expression of E‐cadherin. Yoshikawa *et al*. reported that TSA prevents TGF‐β1‐induced EMT in cultured human renal proximal epithelial cells. However, they did not examine the effect of TSA on N‐cadherin, a representative marker of mesenchymal features 26. Surprisingly, PCI34051, an HDAC8‐selective inhibitor, did not affect E‐cadherin down‐regulation by TGF‐β1 in HK2 cells. However, LMK235, an inhibitor specific to HDAC4 and HDAC5, could partially restore E‐cadherin expression. Therefore, we can conclude that enzyme activity of class I HDACs (HDAC1, 2 and 3), except that of HDAC8, is required for E‐cadherin expression. In addition, class I HDACs rather than class II HDACs are mainly involved in the regulation of E‐cadherin.

Besides the regulation of HDAC enzyme activity, we hypothesized that HDAC expression is associated with the accumulation of ECM or the process of EMT. In the present study, we examined the effect of knockdown of class I HDACs (HDAC1, 2, 3 and 8) on ECM and EMT. Interestingly, knockdown of HDAC1, HDAC2 or HDAC3 did not prevent TGF‐β1‐mediated ECM expression and EMT. This could be attributed to a compensatory effect observed when siRNA against HDAC1 augmented the expression of HDAC2. Conversely, siRNA against HDAC2 elevated HDAC1 protein expression (Fig. 6B) and HDAC3 siRNA transfection increased the protein levels of both HDAC1 and HDAC2. HDAC1 and HDAC2 show high amino acid homology and compensatory functions 27, 28. However, the compensatory effect of HDAC3 siRNA was not reported 29.

In contrast, HDAC8 siRNA did not show a compensatory effect upon siRNA transfection. Indeed, siRNA against HDAC8 successfully suppressed ECM proteins and even significantly restored E‐cadherin in response to TGF‐β1. As addressed above, the HDAC8‐selective inhibitor (PCI34051) did not show the ability to rescue E‐cadherin expression. Taken together, the results suggest that HDAC8 expression itself plays a critical role in the control of E‐cadherin expression rather than HDAC8 enzyme activity. This discrepancy between the effects of the pharmacological inhibitor and siRNA for HDAC8 needs to be further investigated.

In conclusion, we demonstrate the relevance of class I HDACs in regulating ECM proteins and the EMT process *in vitro*. Even though our findings require further confirmatory *in vivo* studies, use of class I‐selective HDAC inhibitors or HDAC8 knockdown could be a useful therapeutic strategy for the treatment of renal fibrosis.

## Conflicts of interest

The authors confirm that there are no conflicts of interest.

## Author contributions

SYC and HJK conceived and designed the experiments. SYC, YR, MQL and GRK performed the experiments. SYC, HJK, LJ and ZHP analysed the data. TK, FKH and MHJ contributed reagents/materials, and reviewed and revised the manuscript. HJK wrote the paper.

## Supporting information


**Figure S1** HDAC2 knockdown did not affect ECM proteins and EMT markers induced by TGF‐β1 in HK2 cells.
**Figure S2** HDAC3 knockdown did not affect ECM proteins and EMT markers induced by TGF‐β1 in HK2 cells.
