# Supplementary material for: Class I HDACs specifically regulate E‐cadherin expression in human renal epithelial cells
Source: J Cell Mol Med. 2016 Jul 15;20(12):2289–98. doi: 10.1111/jcmm.12919 (PMC5134402; doi:10.1111/jcmm.12919)

Supplementary Figure Legends

Supplementary Figure 1. HDAC2 knockdown did not affect ECM proteins and EMT markers induced by TGF-β1 in HK2 cells. (A) For HDAC2 knockdown, HK2 cells were transfected with HDAC2 siRNA or control siRNA. RNA was then extracted and HDAC2 mRNA expression was evaluated using quantitative real-time reverse transcription-polymerase chain reaction (qRT-PCR). $^{**}P < 0.01$ vs. control siRNA. (B) HK2 cells were transfected with HDAC2 siRNA or control siRNA and were then incubated with TGF-β1 for 24 h. The treated cells were then used for western blot analysis. β-actin was used as the loading control. Representative immunoblots for ECM proteins, EMT markers, and class I HDACs are shown. (C-F) Quantification was performed by densitometry for four independent experiments. $^{*}P < 0.05$, $^{**}P < 0.01$, and $^{***}P < 0.001$ compared with control siRNA. NS indicates not significant.

Supplementary Figure 2. HDAC3 knockdown did not affect ECM proteins and EMT markers induced by TGF-β1 in HK2 cells. (A) For HDAC3 knockdown, HK2 cells were transfected with HDAC3 siRNA or control siRNA. RNA was extracted and HDAC3 mRNA expression was evaluated using quantitative real-time reverse transcription-polymerase chain reaction (qRT-PCR). $^{**}P < 0.01$ vs. control siRNA. (B) HK2 cells were transfected with HDAC3 siRNA or control siRNA and were then incubated with TGF-β1 for 24 h. The treated cells were then used for western blot analysis. β-actin was used as the loading control. Representative immunoblots for ECM proteins, EMT markers, and class I HDACs are shown.

(C-F) Quantification was performed by densitometry for four independent experiments. $^{**}P < 0.01$ and $^{***}P < 0.001$ compared with control siRNA. NS indicates not significant.

Supplementary Figure 1

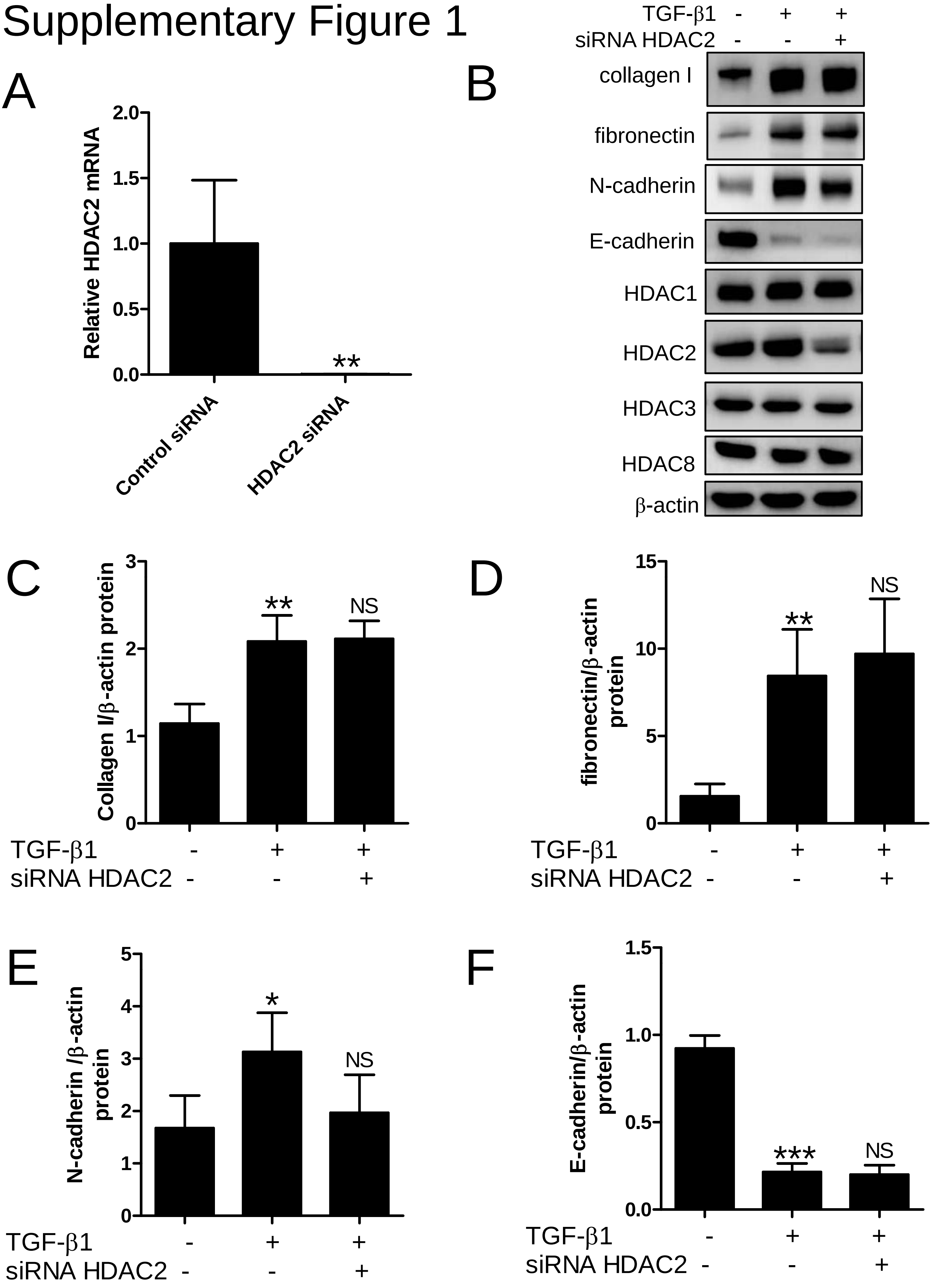

Supplementary Figure 2

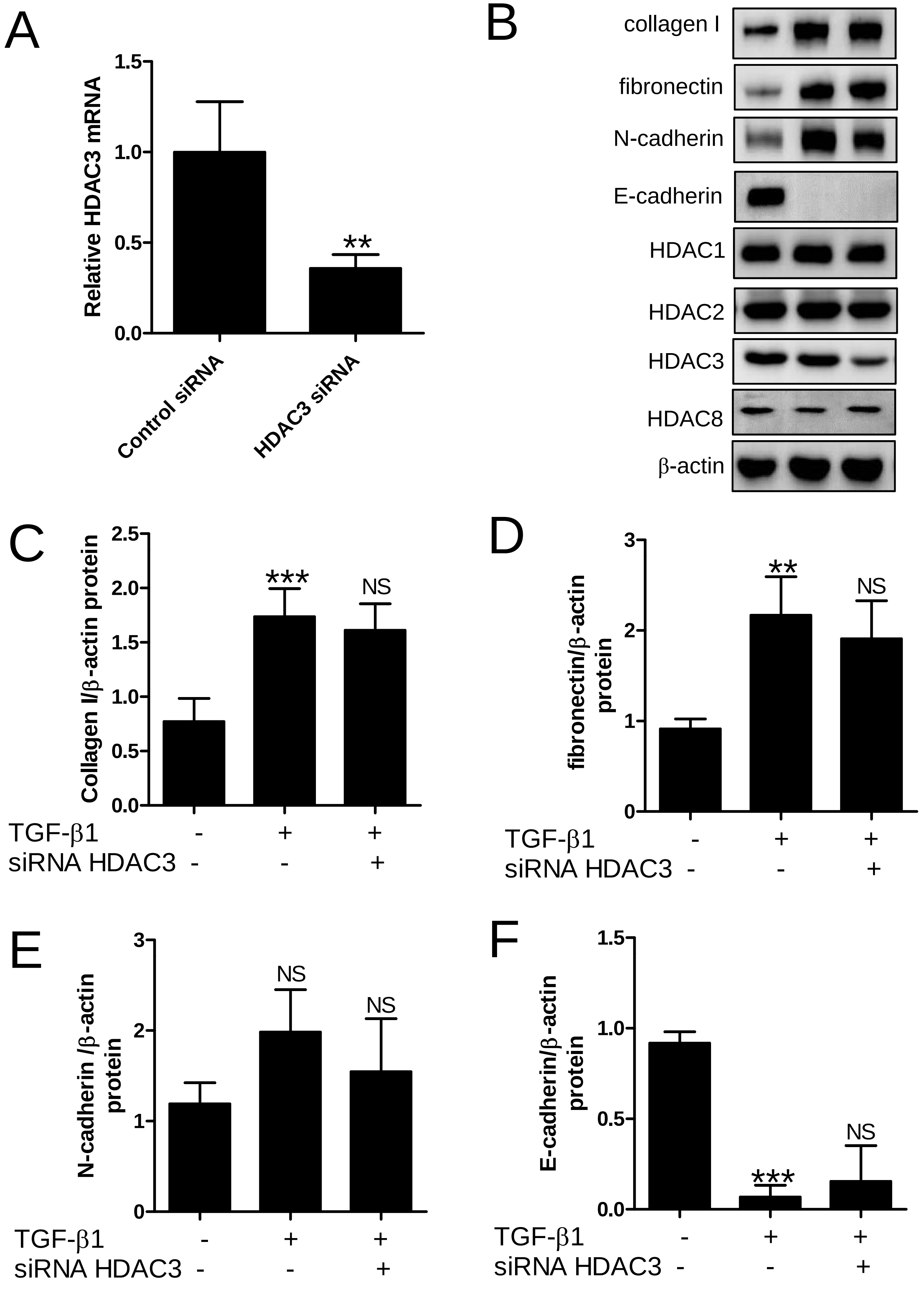

Supplement: Supplementary file 1 — Figure S1 HDAC2 knockdown did not affect ECM proteins and EMT markers induced by TGF‐β1 in HK2 cells. Figure S2 HDAC3 knockdown did not affect ECM proteins and EMT markers induced by TGF‐β1 in HK2 cells. [file JCMM-20-2289-s001.pdf]
